# Delineating the Tes Interaction Site in Zyxin and Studying Cellular Effects of Its Disruption

**DOI:** 10.1371/journal.pone.0140511

**Published:** 2015-10-28

**Authors:** Ermin Hadzic, Marie Catillon, Aliaksandr Halavatyi, Sandrine Medves, Marleen Van Troys, Michèle Moes, Michelle A. Baird, Michael W. Davidson, Elisabeth Schaffner-Reckinger, Christophe Ampe, Evelyne Friederich

**Affiliations:** 1 Laboratory of Cytoskeleton and Cell Plasticity, Life Sciences Research Unit, University of Luxembourg, Luxemburg, Luxembourg; 2 Department of Biochemistry, Ghent University, Ghent, Belgium; 3 National High Magnetic Field Laboratory and Department of Biological Science, The Florida State University, Tallahassee, Florida, United States of America; King's College London, UNITED KINGDOM

## Abstract

Focal adhesions are integrin-based structures that link the actin cytoskeleton and the extracellular matrix. They play an important role in various cellular functions such as cell signaling, cell motility and cell shape. To ensure and fine tune these different cellular functions, adhesions are regulated by a large number of proteins. The LIM domain protein zyxin localizes to focal adhesions where it participates in the regulation of the actin cytoskeleton. Because of its interactions with a variety of binding partners, zyxin has been proposed to act as a molecular scaffold. Here, we studied the interaction of zyxin with such a partner: Tes. Similar to zyxin, Tes harbors three highly conserved LIM domains of which the LIM1 domain directly interacts with zyxin. Using different zyxin variants in pull-down assays and ectopic recruitment experiments, we identified the Tes binding site in zyxin and showed that four highly conserved amino acids are crucial for its interaction with Tes. Based upon these findings, we used a zyxin mutant defective in Tes-binding to assess the functional consequences of abrogating the zyxin-Tes interaction in focal adhesions. Performing fluorescence recovery after photobleaching, we showed that zyxin recruits Tes to focal adhesions and modulates its turnover in these structures. However, we also provide evidence for zyxin-independent localization of Tes to focal adhesions. Zyxin increases focal adhesion numbers and reduces focal adhesion lifetimes, but does so independent of Tes. Quantitative analysis showed that the loss of interaction between zyxin and Tes affects the process of cell spreading. We conclude that zyxin influences focal adhesion dynamics, that it recruits Tes and that this interaction is functional in regulating cell spreading.

## Introduction

The actin cytoskeleton is a highly dynamic cellular system in which actin monomers assemble into filaments that form different actin structures, depending on the cell type and subcellular localization. The actin cytoskeleton is linked to the extracellular matrix through multiprotein complexes called focal adhesions (FAs). FAs play an important role in cellular morphogenesis, proliferation, signaling, cell adhesion and spreading and cell motility. Over 180 proteins are known to participate in the architecture and regulation of FAs [1].

The LIM (Lin-11, Isl-1 and Mec3) domain protein zyxin is one of these proteins. Zyxin localizes to FAs and stress fibers, and is recruited to cell-cell adhesions by nectin [2, 3]. In both structures, zyxin plays an important role in mechanotransduction [4–9]. Upon mechanical stress, zyxin localizes to FAs and recruits Ena/VASP-proteins, which are required to induce a force-dependent actin polymerization [4, 7]. A similar role for zyxin is observed at stress fibers that depend on mechanical forces for their development [7]. Additionally, a zyxin-dependent mechanism for stress fiber repair has been reported [10]. Zyxin is recruited to FAs through its LIM domains, where it has been proposed to play the role of a scaffold protein due to its large number of interactions with other cytoskeleton proteins [11]. The N-terminal region of zyxin harbors an α-actinin binding site, located in the first 50 amino acids, as well as a binding site composed of four proline-rich repeats which are responsible for the interaction with Ena/VASP family members [12–15]. Zyxin and Ena/VASP have been shown to be necessary for the induction of actin polymerization along stress fibers [16]. It has also been proposed that zyxin participates in the regulation of the actin cytoskeleton dynamics by recruiting VASP to FAs and by promoting VASP-dependent actin filament elongation [17, 18]. Furthermore, *zyxin* ^*-/-*^ fibroblasts present enhanced migration and enhanced adhesion compared to wild-type cells, indicating that zyxin plays an important modulatory role in these cellular functions [19]. In addition to α-actinin and Ena/VASP proteins, Tes has been described as a zyxin binding partner in FAs [20, 21].

Similar to zyxin, Tes contains three LIM domains and localizes to cell-cell contacts, stress fibers and FAs [21]. Several studies have highlighted the role of Tes in cell spreading [21–23]. Overexpression of Tes reduces cell motility but enhances cell spreading. In contrast, its knockdown reduces cell spreading and the number of stress fibers and FAs [22]. Moreover, Tes has been proposed to be implicated in the regulation of Mena-dependent cell migration [24]. The overexpression of Tes induces a displacement of Mena from FAs and from the leading edge of the cell to the cytoplasm, leading to a reduction of cell migration speed. Based hereupon, it has been proposed that Tes influences Mena-dependent cell migration by sequestering Mena in the cytoplasm. Similar to zyxin, Tes is capable of interacting with various FA proteins. Through its N-terminal region, Tes interacts with actin, α-actinin and paxillin, whereas the C-terminal region of Tes is responsible for the interaction with Mena, VASP and zyxin [20]. Biochemical analysis has shown that the N-terminal part of Tes is capable of interacting with its C-terminal part, leading to the hypothesis that Tes can adopt two conformations, a closed conformation in the cytoplasm, resulting from the intramolecular interaction, and an open conformation which can be recruited by zyxin to FAs [20]. In FAs, Tes interacts via its LIM1 domain directly with zyxin [20]. However, the interaction site of Tes within zyxin remains unidentified. Furthermore, little is known about the cellular consequences of this interaction.

Here, we have mapped the Tes binding site within zyxin. Based on this, we have generated a zyxin mutant which is unable to interact with Tes. We subsequently used this mutant to gain insight in zyxin-dependent recruitment of Tes to FAs. Performing fluorescent recovery after photobleaching (FRAP), we have demonstrated that the interaction of Tes with zyxin stabilizes Tes in FAs. Furthermore, we have shown that zyxin regulates FA dynamics independently of Tes, whereas the interaction between the two proteins promotes cell spreading.

## Materials and Methods

### Plasmid constructs and siRNA oligonucleotides

Tes FL-GFP was generated by cloning a cDNA corresponding to the full length protein (residues 1–421) into a pEGFP-N3 vector (Clontech). Tes LIM cDNA (coding for residues 234–421) and Tes-LIM1 cDNA (coding for residues 234 to 299) were generated by PCR using Tes FL-GFP vector as a template, and cloned into pEGFP-C3 vectors (Clontech). To generate the GST tag construct, Tes LIM1 was amplified by PCR and cloned into a pGEX4T2 vector (Clontech). To generate GFP-tagged zyxin variants, the cDNA corresponding to Zyx FL WT (1–572), Zyx FL MT (1–572), Zyx 51–63 and Zyx 51-63-MT were cloned into pEGFP-N3 vector (Clontech). Zyx FL WT-DsRed and Zyx FL MT-DsRed were generated by substituting the EGFP gene by a DsRed gene. Zyx FL WT-mito (1–572) and Zyx FL MT-mito (1–572), Zyx NT-mito (1–380), Zyx LIM-mito (332–572), and the shorter zyxin variants (140–380), (51–140), (51–110), (111–140), (51–92), (51–77), (51–63 WT) and (51–63 MT) were cloned into the pUHD10-3 vector which encodes a Myc (9E10) tag followed by a mito tag, except for the two Zyx FL (WT and MT) constructs which lack the Myc tag. The mito tag inserts itself into the mitochondrial membrane, thus allowing to target fusion proteins to the surface of mitochondria. A sequence of zyxin in which the codons of the four amino acids 60VGEI63 were substituted by four alanine codons was synthesized by DNA2.0. This sequence was used for generating the zyxin variants in the pUHD10-3, pEGFPN3 and pDsRed vectors. All constructs were verified through sequencing by LGC Genomics.

Small interfering RNA oligonucleotides, directed against Tes or zyxin, were purchased from Qiagen. The siRNA sequence targeting Tes corresponds to: AACTACACTTCTGGAGGAAAA. For zyxin knockdown the following siRNA sequence was used: AAGTGTTACAAGTGTGAGGAC.

### Cell culture and transfection

Vero monkey kidney cells (ATCC CCL-81), HeLa cells (ATCC: CCL-2), mouse embryonic fibroblasts (MEF, ATCC: SCRC-1008) and zyxin-null mouse fibroblasts were grown at 37°C and under 5% CO_2_ in Dulbecco’s modified Eagle’s medium (DMEM) (Lonza) supplemented with glutamine, penicillin/streptomycin (Lonza) and 10% fetal calf serum (Lonza). Electroporation of Vero cells was carried out with a total of 15 μg plasmid for 5×10^6^ cells at 240 V and 950 μF. HeLa cells were transfected by phosphate-calcium method as previously described [25]. Wild-type (MEF) and zyxin-null fibroblasts were transfected with lipofectamine 2000 (Invitrogen) according to the manufacturer’s protocol. All experiments were carried out 24 hours post transfection.

### Antibodies and fluorochrome-coupled probes

For immunofluorescence staining, Myc tagged zyxin variants were detected using a monoclonal antibody (clone 9E10, Life Technologies) recognizing the Myc epitope (VAACNMEQKLISEEDLNMNS) (Golsteyn et al., 1997). Endogenous zyxin and the constructs Zyx FL-mito and Zyx FL MT-mito were detected in cells with a polyclonal zyxin antibody, obtained from the immunization of a rabbit with two peptides located in the N-terminal region of zyxin (Eurogentec). Endogenous vinculin was visualized using a specific monoclonal mouse antibody (clone VIN-11-5, Sigma-Aldrich). Tes was detected using a polyclonal rabbit antibody raised against the N-Terminal domain of Tes (Eurogentec). VASP was revealed with a monoclonal mouse antibody (clone 43/VASP, Transduction Laboratories). α-actinin was stained with a monoclonal mouse antibody (clone BM-75.2 SIGMA) and F-actin was labeled with Alexa Fluor 594 phalloidin (Molecular Probes). Secondary IgG antibodies directed against mouse or rabbit were coupled to Alexa Fluor dyes (Invitrogen).

For Western blot detection, polyclonal anti-zyxin, monoclonal anti-GFP (clone GFP-20, Sigma-Aldrich), polyclonal anti-GST (Cat.# 06–332, Upstate), a monoclonal anti-Tes (clone G9, Santa Cruz Biotechnology) or monoclonal anti-β-actin (clone Ac-15, Sigma-Aldrich) antibodies were used. A secondary antibody coupled to horseradish peroxidase (Amersham, Biosciences) was added and the protein bands were revealed with Super Signal WEST FEMTO (Thermo Scientific), using a ChemiLux imager system (Intas Science Imaging). Alternatively, protein bands were revealed based on fluorescence using secondary antibodies coupled to a fluorescent dye and an Odyssey Infrared imaging system (Licor Biosciences). In this case, donkey anti-mouse IRdye® 680 (Licor Biosciences) or goat anti-rabbit IgG DyLight 800 (Thermoscientific) were used.

### Production of recombinant proteins and GST pull-down

The recombinant protein GST-Tes LIM1 was produced in *Escherichia coli* strain BL21, purified from the soluble fraction using a Glutathione Sepharose 4B resin (GE Healthcare) and was eluted using a reduced glutathione buffer (50 mM Tris pH 8; 10 mM reduced glutathione). The purified recombinant proteins were quantified by Bradford method (Bio-rad) and analyzed on denaturing SDS-PAGE and stored at -80°C. For GST pull-down assays, the glutathione Sepharose 4B resin was washed three times with NP40 lysis buffer (50 mM Tris pH 7.4; 120 mM NaCl; 0.5% Nonidet P-40; 0.5 mM EDTA). 16 μg of the purified GST-Tes LIM1 variant were immobilized on 80 μl Glutathione Sepharose 4B resin (slurry 50%) and incubated during 1 hour using continuous rotation at 4°C. The non-bound fraction was removed by centrifugation. The fixation of the GST recombinant proteins on beads was verified on SDS-PAGE. HeLa cells were transfected with the corresponding GFP constructs and lysed 24 hours after transfection with the NP40 lysis buffer containing protease inhibitor cocktail (ROCHE) and total protein concentrations were measured by Bradford method (Bio-rad). 80 μl of beads (slurry 50%) with immobilized GST recombinant proteins were incubated with 200 μg of total proteins during 2 hours using continuous rotation at 4°C. After centrifugation at 2000 g during 5 minutes at 4°C, 18 μl of the non-bound fraction (NB) were collected. Beads were washed extensively in NP40 lysis buffer. After removing the supernatant, the beads corresponding to the bound fraction (B) were suspended in 18 μl of SDS-Page buffer. For each condition, bound fraction (B) and non-bound fraction (NB) were loaded on denaturing SDS-PAGE and analyzed by Western blot.

### Microscopy

Fixed cells were processed for immunofluorescence staining as previously described [17], and were imaged with an epifluorescence microscope (Leica DMRX, HCX PL APO 63x/1.32NA or 100x/1.35NA oil immersion lenses) equipped with the appropriate excitation and emission filters. Images were acquired with a linear CCD camera (Micromax; Princeton instruments) and Metaview software (Universal Imaging). All confocal microscopy experiments with living cells and fixed samples were performed with a Zeiss LSM 510 Meta laser scanning confocal microscope (Carl Zeiss, Jena, Germany) using objectives and acquisition settings specified below. Living cells were maintained on the microscope stage at 37°C in a 5% CO_2_ atmosphere using an air-stream incubator and a heating stage insert (Pecon GmbH). Images were analyzed with custom ImageJ plugins (NIH) as described below.

### Cell spreading assay

Fibronectin-coated coverslips were prepared by incubating them with 20 μg/ml of fibronectin in PBS (140 mM NaCl, 2.7 mM KCl, 6.5 mM Na_2_HPO_4_, 1.5 mM KH_2_PO_4_, pH 7.2), followed by saturation at 37°C for 1 hour with 1% BSA diluted in PBS. Zyxin-null fibroblasts were transfected as indicated. 24 hours after transfection cells were washed with PBS and trypsinized. The cells were resuspended for 30 minutes and then plated onto the fibronectin-coated coverslips. After incubation for 15, 30, 60 or 240 minutes at 37°C and 5% CO_2_, cells were washed twice with PBS, fixed with 3% PFA for 20 minutes and stained with Alexa Fluor 594 phalloidin (Molecular Probes). After staining, multiple fields were imaged using the confocal microscope with a Plan-Apochromat 20x/0.8NA dry lens. To quantify cell areas we first subjected images in both channels to background subtraction and to filtering with a low-pass filter. Cells were segmented with a constant intensity threshold level. To select transfected cells the matches between segmented regions in green and in red channels were identified. Automated analysis was manually verified to exclude segmented regions that contained more than one cell or cells with abnormal expression levels. For each condition at least 300 cells were analyzed. Statistical significances for the differences between mean cell areas were estimated using 1-way ANOVA followed by Tukey’s significant difference test to compare each pair of conditions.

### Quantification of FAs and FA proteins

Zyxin-null fibroblasts were transfected with Tes FL-GFP and DsRed, Zyx FL WT-DsRed or Zyx FL MT-DsRed and were fixed 24 hours after transfection. Endogenous vinculin was stained with a mouse monoclonal anti-vinculin and an Alexa Fluor 647-coupled secondary antibody. For actin quantification, zyxin-null fibroblasts were transfected with DsRed, Zyx FL WT-DsRed or Zyx FL MT-DsRed, vinculin was labelled with the same primary antibody and an Alexa Fluor 488-coupled secondary antibody. Similarly, for siRNA experiments, cells were stained for vinculin and actin using the same antibodies and phalloidin probes. Imaging was performed with the confocal microscope in square regions of 1024×1024 pixels (0.14 μm/pixel) with a Plan-Apochromat 63x/1.4NA oil-immersion objective and a confocal pinhole set to 122 μm. Fusion protein expression levels were taken into account and only cells with similar levels of expression were selected. To segment FAs and to quantify the corresponding fluorescent signals we applied a customized version of a previously reported algorithm [26]. Images in vinculin channel were subjected to background subtraction with the rolling-ball algorithm and used to segment FAs, minimal FA size was set to 20 pixels (0.39 μm^2^). Fluorescence signals in all channels were quantified in segmented regions using non-transformed images. To account for fluorescence that comes from non-bound proteins, for each FA we calculated the average intensity of pixels which are in the vicinity of the selected FA (closer than 4.2 μm to its centroid), but which do not belong to any FA and which are located within the analyzed cell. When this value was subtracted from the average intensity within FA, the result was multiplied by FA area to estimate the total amount of the bound protein. Measured FA sizes and protein quantities were averaged for each cell and these numbers were used to build the final bar plots (represented as mean ± S.E.M. for each condition) and to perform statistical tests. Statistical significances for the differences of mean parameters for each pair of conditions were estimated using 1-way ANOVA followed by Tukey’s significant difference test, or t-test when two conditions were compared.

### Quantification of FA turnover rates

Zyxin-null fibroblasts were transiently co-transfected with mEmerald-paxillin and DsRed, Zyx FL WT-DsRed or Zyx FL MT-DsRed. Time series of 60 minutes (30 seconds time intervals) were acquired on the confocal microscope with a Plan-Apochromat 63x/1.4NA oil-immersion lens and a confocal pinhole set to 300 μm. mEmerald paxillin was detected using 488 nm Argon laser line and a 505 to 550 nm band-pass emission filter. DsRed was detected with a DPSS-561 laser and a 575 nm long-pass emission filter. In each case imaging was performed with 2% of maximum laser power, a minimum of 10 cells were analyzed for each condition. Only cells with similar levels of expression were selected. To track FAs through their lifetime, the cell in question was segmented using the mEmerald channel and all FAs within this cell were identified at each time step similarly to what was done for the quantification of FAs in fixed cells. Lineages of the segmented FAs were identified following the rules described in [27]. Lineages which are shorter than 3 sequential time points were discarded. The remaining tracks were used for analysis (3040, 4553 and 3013 tracks for Control, Zyx FL WT and Zyx FL MT respectively) To calculate the distribution of FA lifetimes we considered only FAs which appeared as new objects and completely disassembled during the observation period (1023, 1618 and 1006 tracks for Control, Zyx FL WT and Zyx FL MT respectively). The number of FAs within each subgroup on the lifetime histogram was normalized to the total number of FAs selected for the analysis of lifetimes. We considered FAs as being stable if they were present at the start of the acquisition and existed throughout the observation period (25, 65 and 51 tracks for Control, Zyx FL WT and Zyx FL MT respectively). For comparison, these numbers were normalized to the average number of FAs detected per time point for each condition. To calculate p-values for the differences between the fractions of FAs within each subgroup of the lifetime distributions and between the fractions of stable FAs we used pairwise z-test for proportions with Benjamini-Hochberg correction.

### Fluorescence recovery after photobleaching

FRAP experiments were performed on the confocal microscope with a Plan-Apochromat 63x/1.4NA oil-immersion lens and a confocal pinhole set to 300 μm. GFP-labelled zyxin variants and Tes FL-GFP were detected with 488-nm Argon laser line and 505 to 550 nm band-pass emission filter, mCherry-actin was detected using a DPSS-561 laser and a 575 to 615 nm band-pass emission filter. In each case imaging was performed with 2% of the maximum laser power. The same laser line was used for bleaching fluorochromes within circular spots with a diameter of 2.7 μM covering FAs. Three bleaching iterations were performed in less than 0.2 seconds with the laser power adjusted to bleach either 100% (for Tes and actin) or about 50% (for zyxin variants) at FAs. FRAP acquisitions were performed in rectangular regions of 512×128 pixels (0.14 μm/pixel). In FRAP experiments with Tes FL-GFP and with GFP-labelled zyxin variants, 20 images before bleaching and 220 images after bleaching were acquired with time intervals between images equal to 0.5 seconds. In experiments with mCherry-actin, the time-lapse images were simultaneously acquired in two fluorescent channels to use zyxin GFP variants as a marker for the subsequent tracking of FAs. Twenty images before bleaching and 400 images after bleaching were acquired with the time interval between images equal to 1 second. To properly account for FA sliding observed in zyxin-null fibroblasts, the movement of the bleached FAs was tracked with the algorithm used for quantification of FA lifetimes (only one FA per time series tracked in FRAP experiments). Average fluorescence intensities inside the identified FA masks were calculated at each time point. Quantified FRAP curves were normalised using Eq (1) to correct for background fluorescence, acquisition photobleaching and laser fluctuations [28, 29].

$$F(t)=\frac{I_{frap}(t)-I_{base}(t)}{I_{ref}(t)-I_{base}(t)}\cdot \frac{I_{ref\_pre}}{I_{frap\_pre}}$$ (1)

where *I*_*frap*_(*t*) - quantified intensity in the bleached region; *I*_*ref*_(*t*) - average intensity of the cell; *I*_*base*_(*t*) - background intensity measured out of the cell; subscript _pre means the averaging of intensities for the prebleach time points after background subtraction. Depending on the experiment, at least 80 (Tes and Zyx) or 15 (actin) recovery curves per condition, collected during 4 independent experiments, were analyzed. Each normalised FRAP curve was fitted separately with Eq (2):

$$F_{fit}(t)=I_{0}-I_{1}e^{-k_{t}t}$$ (2)

where *I*_0_ - normalised fluorescence plateau at the end of recovery; *I*_1_ - amplitude of the recovery; *k*_*t*_ - the turnover rate that is inversely proportional to the recovery halftime (t12=ln(2)kt).

The estimated recovery halftimes are represented on a logarithmic scale as Box-and-Whisker plots overlaid with individual data points and thin lines inside boxes representing mean recovery halftimes for each condition. Because the distribution of the FRAP recovery halftimes deviated significantly from normal distribution, we used non-parametric methods to evaluate these data. The Mann-Whitney U test (2 conditions for FRAP with zyxin and with actin) or Kruskal-Wallis test followed by pairwise Wilcoxon test with Benjamini-Hochberg correction (3 conditions for FRAP with Tes) were used to test for the statistical significance of the increase or decrease of the estimated parameters. To compare variations of Zyx FL WT and Zyx FL MT recovery halftimes, we calculated logarithm of each estimated halftime value and applied Fligner-Killeen test of homogeneity of variances on these values. The logarithmic transformation resulted in the parameters distributions that were much closer to normal in comparison with initial distributions and equalized the impact of high and low FRAP halftime values in this statistical test.

## Results

### Tes interacts with the N-terminal region of zyxin

**Fig 1:**
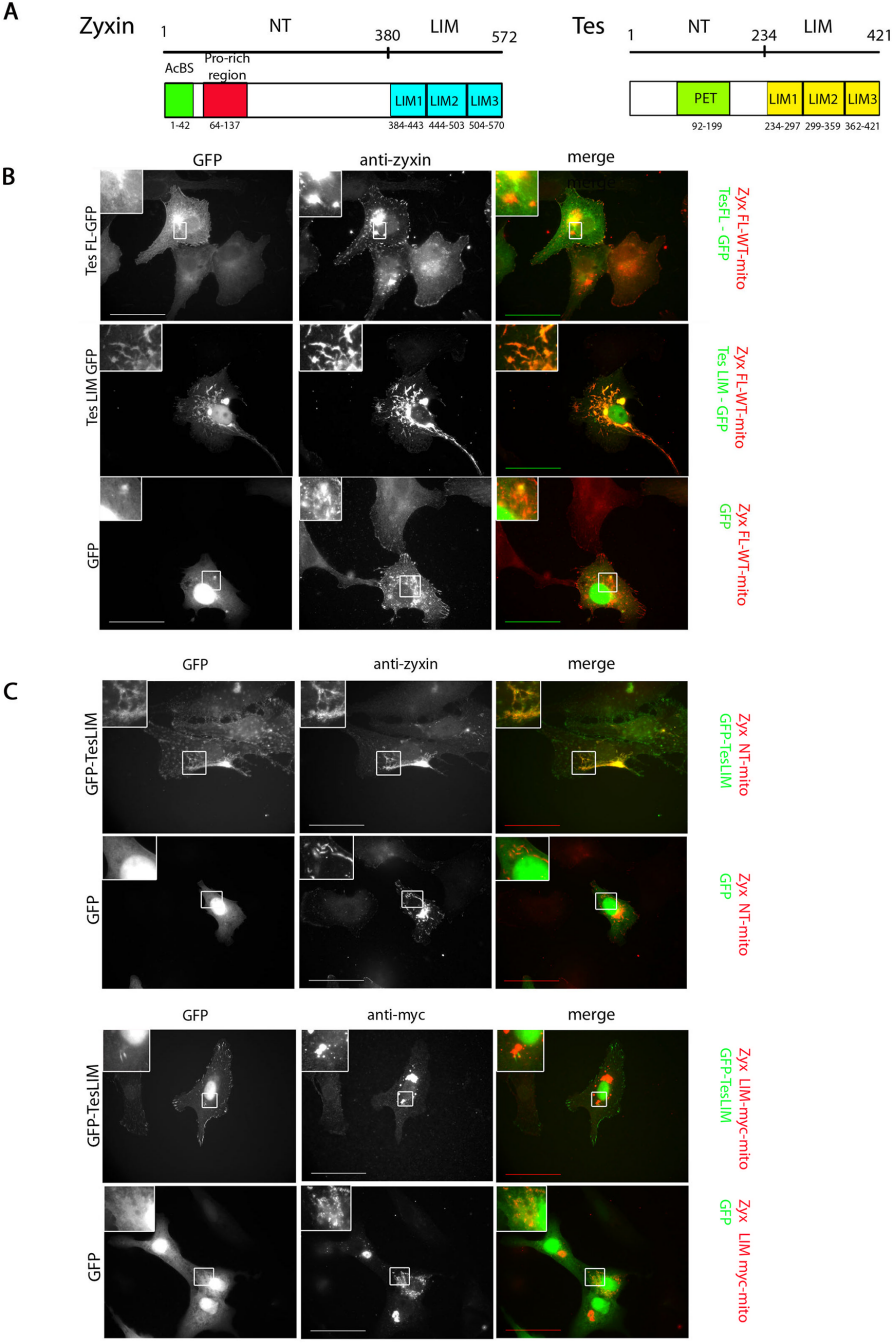
Ectopic recruitment to mitochondria of Tes and Tes LIM by zyxin-mito variants. (A) Schematic representation of the LIM domain proteins zyxin and Tes. The α-actinin binding site (AcBS) and the proline-rich region binding Ena/VASP family members (Pro-rich region) of zyxin are shown as well as the LIM domains. For Tes, the PET domain in the N-terminal part and the three LIM domains in the C-terminal part are depicted. In (B) and (C), Vero cells were transiently transfected with Zyx-mito variants and Tes FL-GFP, GFP-Tes LIM or GFP (control). Zyx FL WT-mito (B) and Zyx NT-mito (C) were labelled with an anti-zyxin antibody and Zyx LIM-myc-mito was labelled with an anti-myc antibody. Scale bar: 50 μm; the insets show a higher magnification of the outlined regions.

Previous studies have shown that zyxin regulates the localization of Tes by recruiting Tes to FAs [20]. Furthermore, it was shown that the first LIM domain of Tes (Fig 1A) interacts directly with zyxin. So far, the binding site of Tes within zyxin remained unknown. We performed ectopic recruitment experiments on the surface of mitochondria as well as pull-down assays to analyze the capacity of different zyxin variants to interact with Tes and to map and identify the Tes binding site in zyxin.

As illustrated in Fig 1B, the full-length protein Tes FL-GFP showed only a weak colocalization with the wild-type zyxin, Zyx FL WT-mito, that is targeted to the surface of mitochondria by the membrane anchor of the *Listeria monocytogenes* protein ActA (mito). In contrast, Tes LIM-GFP, which corresponds to the 3 LIM domains of Tes (amino acids 234–421) was strongly recruited to the mitochondrial surface by Zyx FL WT-mito, consistent with published data that zyxin likely interacts with the open conformation of Tes [20]. Additionally, as Tes LIM-GFP interacted with Zyx NT-mito but not with Zyx LIM-myc-mito (Fig 1C), our results suggest that it is the N-terminal region of zyxin that mediates the interaction with the LIM domains of Tes.

### Delineation of the Tes-binding site in the N-terminal region of zyxin

**Fig 2:**
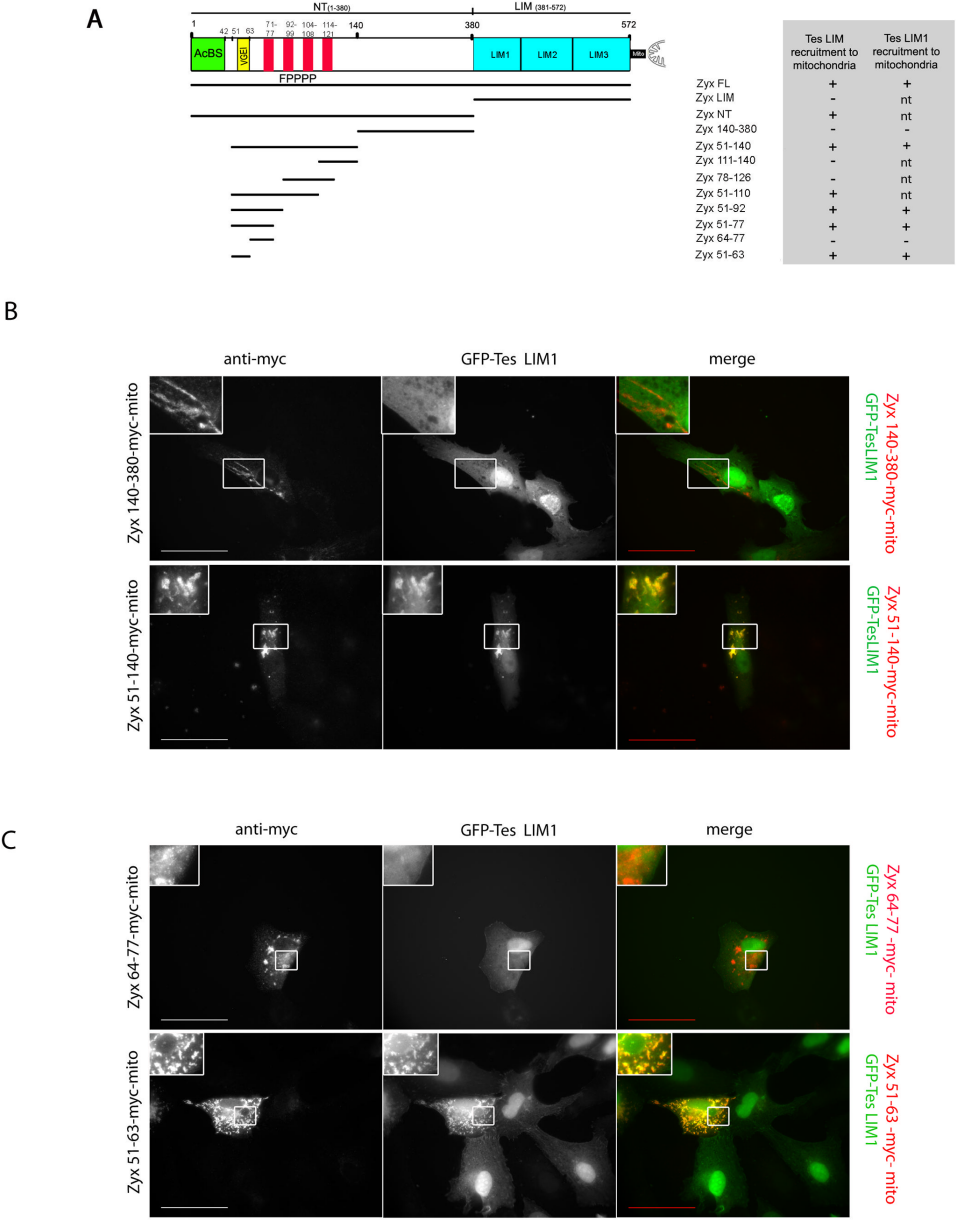
Determination of the minimal sequence of zyxin that interacts with Tes. (A) Schematic representation of zyxin (1–572), fused to the mitochondrial targeting sequence (mito). The green box corresponds to the α-actinin binding site (AcBS), the four red boxes correspond to the four FPPPP motifs binding VASP, the yellow box represents the amino acids VGEI. Zyxin-mito variants are represented by lines showing their respective position in the full-length protein (in the same order as in the inset table). Their ability to recruit GFP-Tes LIM and GFP-Tes LIM1 on the surface of mitochondria in Vero cells is indicated in the table (nt: not tested). (B) and (C) Ectopic recruitment of GFP-Tes LIM1 by the zyxin variants Zyx 140-380-myc-mito, Zyx 51-140-myc-mito, Zyx 64-77-myc-mito and Zyx 51-63-myc-mito to the surface of the mitochondria in Vero cells. The different zyxin variants were labelled with an anti-myc antibody. Scale bar: 50 μm; the insets show a higher magnification of the outlined regions.

The N-terminal region of zyxin contains the binding site for α-actinin [15] as well as four FPPPP motifs necessary for interaction with members of the Ena/VASP family [13, 16, 17] (Fig 2A). Knowing that Tes is also capable to interact with α-actinin, VASP and Mena, the mitochondrial recruitment observed above could result either from a direct recruitment or from an indirect recruitment of Tes to zyxin mediated by these partners. To address this issue and further map the sequences of zyxin that are implicated in the direct recruitment of Tes, we first generated two myc-mito variants of Zyx NT; a variant lacking the proline rich sequences (Zyx 140-380-myc-mito) and a variant containing the proline rich sequences but lacking the α-actinin binding site (Zyx 51-140-myc-mito). Immunofluorescence analysis of the subcellular distribution of these zyxin variants revealed that Zyx 140-380-myc-mito failed to recruit GFP-Tes LIM or GFP-Tes LIM1 on the surface of the mitochondria (Fig 2A table inset and Fig 2B) suggesting that the region between the proline rich sequences and the LIM domains is not implicated in the interaction with Tes. In contrast, the variant Zyx 51-140-myc-mito retained the capacity to recruit GFP-Tes LIM or GFP-Tes LIM1 to the mitochondria (Fig 2A table inset and Fig 2B). This additionally indicates that the interaction between Tes and zyxin is independent from α-actinin. To further dissect this region, we generated variants corresponding to parts of the sequences in region 51–140: Zyx 51–110, 51–92, 51–77, 78–126 and 111–140. Subsequent analysis revealed that the variant Zyx 51-77-myc-mito in this series was the smallest peptide able to recruit GFP-Tes LIM (containing the three LIM domains) or GFP-Tes LIM1 (containing only the first LIM domain with the zyxin binding site) (Fig 2A table inset). The sequence enclosed in residues 51–77 contains 13 residues (amino acids 51–63) followed by the first proline rich region of zyxin (amino acids 64–77) containing the first FPPPP motif. Thus, we designed two smaller variants corresponding to residues 51–63 (Zyx 51-63-myc-mito) and 64–77 (Zyx 64-77-myc-mito), respectively. Contrary to residues 64–77, residues 51–63 recruited GFP-Tes LIM and GFP-Tes LIM1 (Fig 2A table inset and Fig 2C). These results show that the Tes LIM1 binding site in zyxin is localized in the region 51–63 and is independent of the FPPPP motifs (and thus independent of recruitment by members of the Ena/VASP family) as well as of the α-actinin binding site.

### The four amino acids VGEI in zyxin are necessary for the interaction with Tes

**Fig 3:**
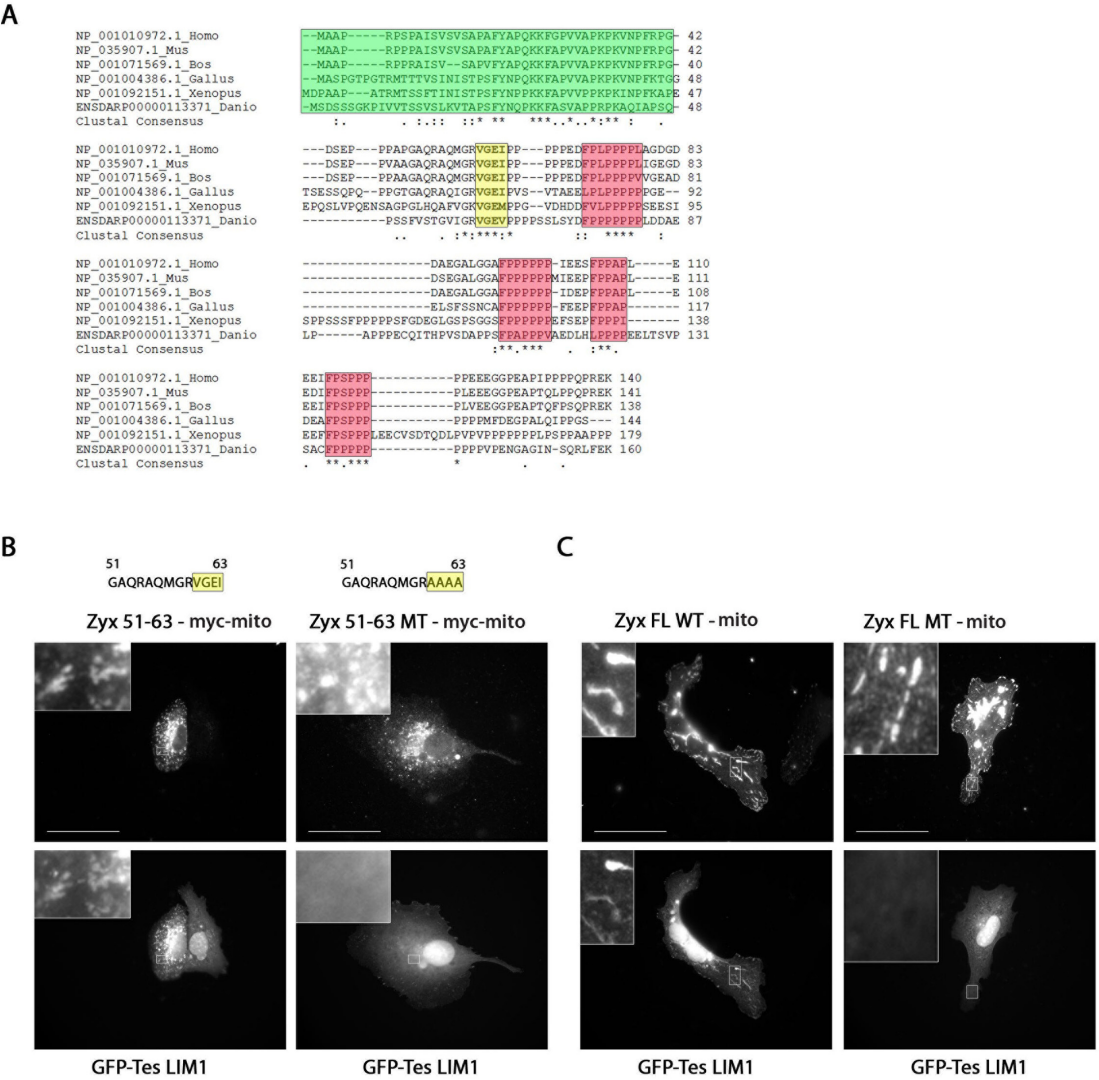
The sequence 51–63 contains 4 conserved amino acids VGEI important for the interaction between Tes and zyxin. (A) Sequence alignment of the first 136 amino acids of human zyxin with orthologues (reference sequences with the indicated numbers were retrieved from the NCBI protein database for the indicated species, the zebrafish sequence was retrieved from Ensembl). Protein sequences were aligned with ClustalW2. The α-actinin binding site (green), the VGEI sequence (yellow) and the FPPPP motifs (red) are shown. (*) represents identical residues, (:) represents conserved substitutions and (·) represents semi-conserved substitutions in the alignment. (B) and (C) Ectopic recruitment of GFP-Tes LIM1 by zyxin variants to the surface of mitochondria in Vero cells. (B) Zyx 51-63-myc-mito and Zyx 51–63 MT-myc-mito sequences are indicated on top, both variants were labelled with an anti-myc antibody (top panels), (C) Zyx FL WT-mito and Zyx FL MT-mito were labelled with an anti-zyxin antibody (top panels). Bottom panels show GFP-Tes LIM1 signals. Scale bar: 50 μm; the insets show a higher magnification of the outlined regions.

Protein interaction sites are often strongly conserved among homologues. To evaluate this for the identified Tes binding site in zyxin, we performed protein sequence alignments of zyxin proteins from different species. Interestingly, the alignments showed that residues 51–63 are strongly conserved among species, especially in the C-terminal part (Fig 3A). We mutated the sequence 60VGEI63 in this fragment of zyxin into four alanines (Zyx 51–63 MT-myc-mito, MT indicates mutant carrying 60AAAA63). Ectopic recruitment experiments showed that this mutant fragment failed to recruit GFP-Tes LIM1, in contrast to the VGEI containing fragment Zyx 51-63-myc-mito (Fig 3B). We reproduced this mutation in full length zyxin (Zyx FL MT-mito). Similar to the Zyx 51–63 MT-myc-mito construct, Zyx FL MT-mito lost the capacity to recruit GFP-Tes LIM1 (Fig 3C). Similarly to Zyx FL WT, Zyx FL MT retained the capacity to bind and recruit α-actinin as well as VASP (S1 Fig), indicating that these two binding sites, which flank the Tes binding site on both sides, were not affected by the mutation.

**Fig 4:**
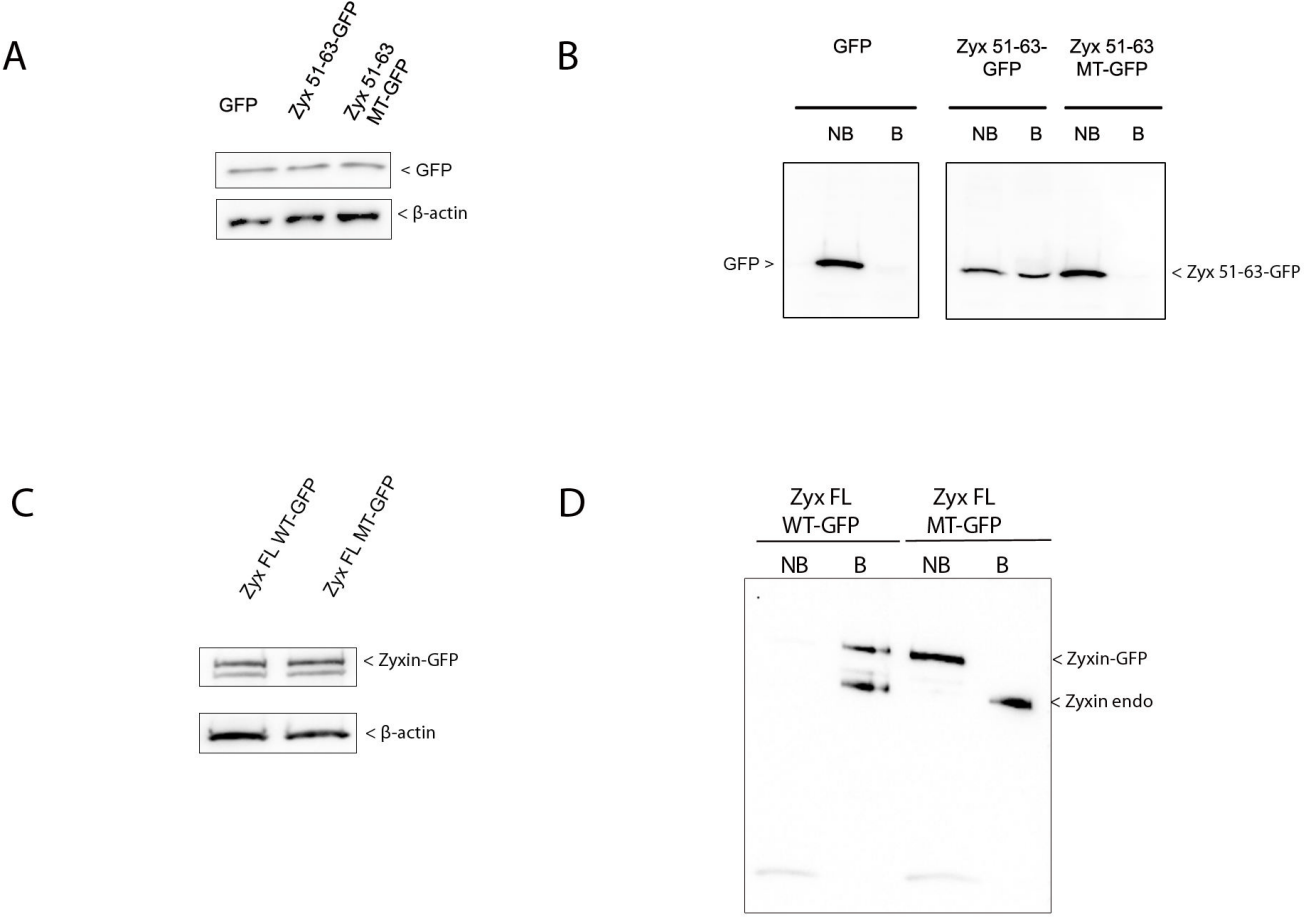
The VGEI sequence in full-length zyxin is necessary for the interaction with Tes LIM1. (A) and (C) expression levels of GFP-fusion proteins in HeLa cells were verified by Western blot using an anti-GFP antibody (A), an anti-zyxin antibody (C) and an anti-β-actin antibody (control) (A, C). (B) Western blot analysis of GST pull-down experiments performed with GST-Tes LIM1 immobilized on glutathione sepharose resin, and extracts of HeLa cells transfected with Zyx 51-63-GFP, Zyx 51–63 MT-GFP or GFP (negative control). The presence of zyxin-GFP variants was analyzed in bound “B” or non-bound “NB” fractions using an anti-GFP antibody. (D) Zyx FL WT-GFP and Zyx FL MT-GFP extracts from transfected HeLa cells, were analyzed in bound (B) and non-bound (NB) fractions using an anti-zyxin antibody. Note the presence of endogenous zyxin (zyxin endo) in the bound fraction.

To biochemically confirm the importance of the VGEI sequence, we performed pull-down assays with GST-Tes LIM1 (amino acids 234–299) produced in *E*. *coli* and zyxin-GFP fusion proteins from HeLa cell extracts (Fig 4A and 4C, showing equal expression levels of zyxin WT and MT). Western blot analysis using a GFP-specific antibody revealed the interaction between Tes LIM1 and Zyx 51-63-GFP. However, in the case of the variant Zyx 51–63 MT-GFP, no interaction could be detected (Fig 4B). We were able to pull down Zyx FL WT-GFP as well as endogenous zyxin with GST-Tes LIM1, this in contrast to Zyx FL MT-GFP (Fig 4D).

**Fig 5:**
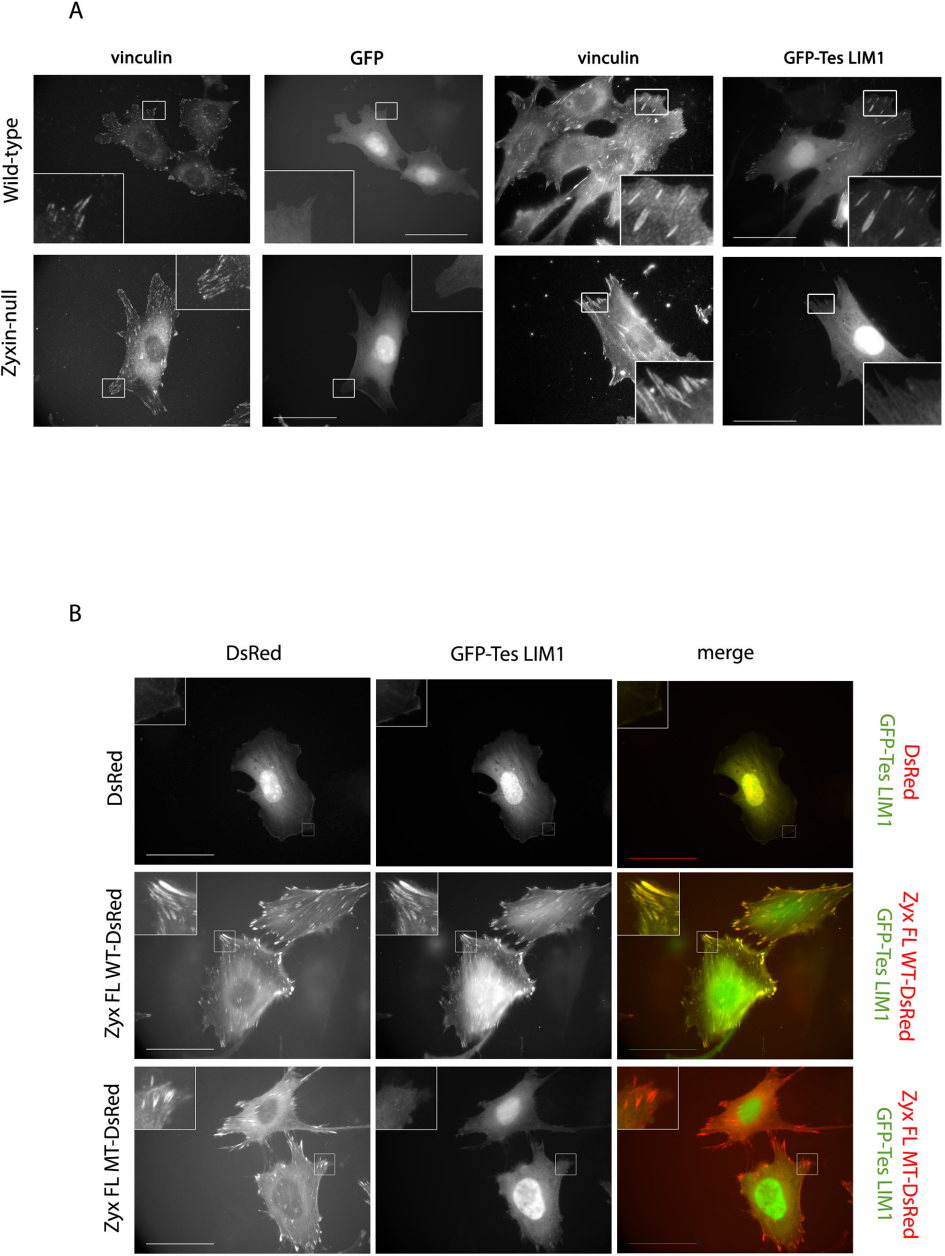
The recruitment of Tes LIM1 to FAs depends on the VGEI sequence of zyxin. (A) Wild-type and zyxin-null fibroblasts were transfected with GFP-Tes LIM1 or GFP (control). FAs were stained with an anti-vinculin antibody. (B) Zyxin-null fibroblasts were cotransfected with GFP-Tes LIM1 and DsRed, Zyx FL WT-DsRed or Zyx FL MT-DsRed. Scale bar: 50 μm, the insets show a higher magnification of the outlined regions.

To determine whether this Tes interaction site is functional in FAs, we used fibroblasts in which the zyxin gene was deleted by homologous recombination (further referred to as zyxin-null) [19]. In wild-type fibroblasts, GFP-Tes LIM1 localized to FAs in contrast to zyxin-null fibroblasts in which GFP-Tes LIM1 failed to accumulate to FAs (Fig 5A). We compared the ability of the Zyx FL WT and Zyx FL MT to recruit Tes, via its LIM1-domain to FAs, by co-expressing these forms as DsRed fusions with GFP-Tes LIM1 in zyxin-null fibroblasts. In contrast to Zyx FL WT-DsRed, the mutant Zyx FL MT-DsRed did not exhibit the capacity to recruit GFP-Tes LIM1 to FAs (Fig 5B). Taken together, these findings demonstrate that the amino acids 60VGEI63 in zyxin are necessary for the interaction with Tes at FAs, in particular with its LIM1 domain.

### Tes can be recruited to focal adhesions in a zyxin-independent manner

**Fig 6:**
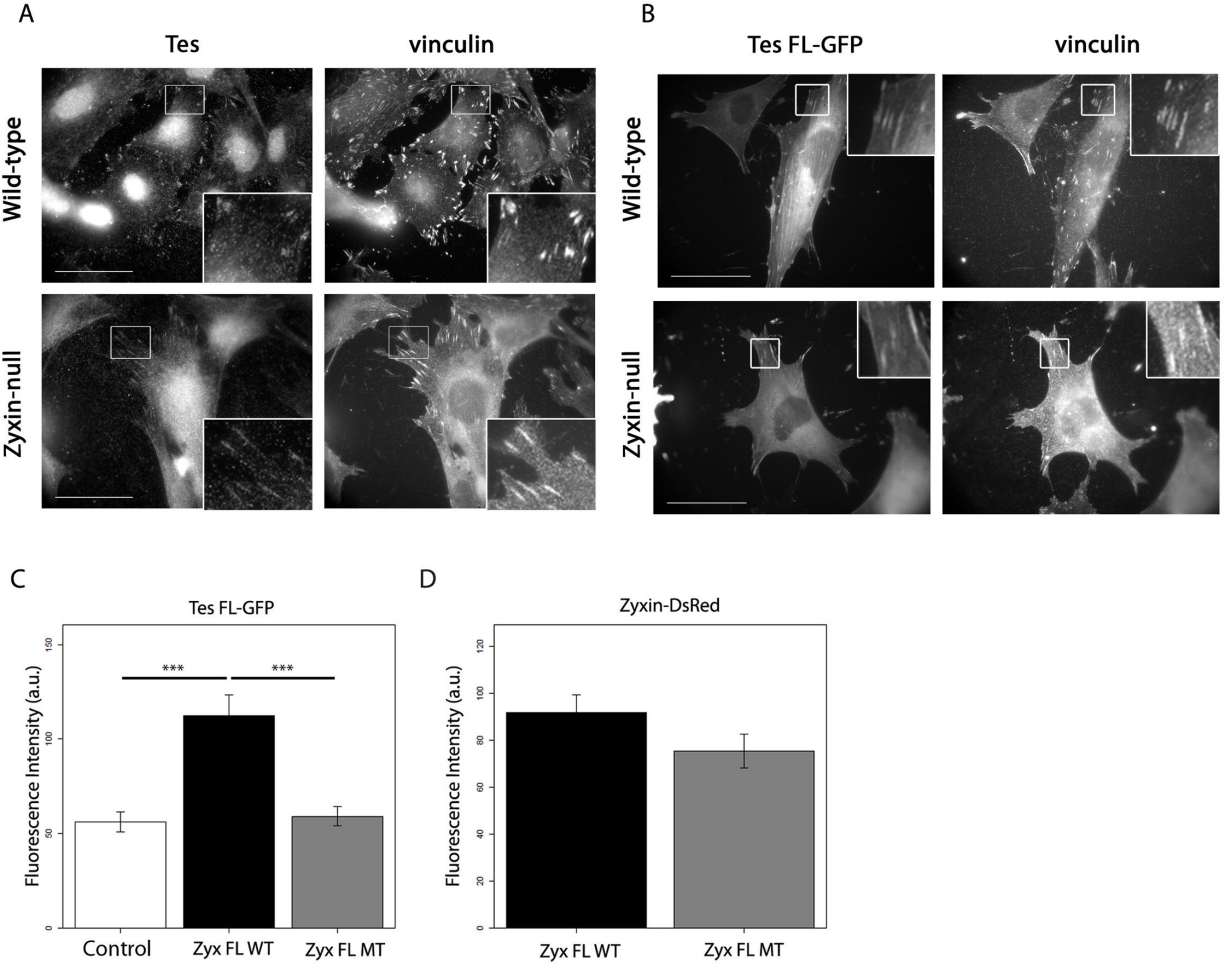
Tes localizes to FAs in the absence of zyxin. (A) Endogenous Tes was stained with an anti-Tes antibody in wild-type and zyxin-null fibroblasts and the FA marker vinculin was revealed with an anti-vinculin antibody. (B) Wild-type and zyxin-null fibroblasts were transfected with Tes FL-GFP and stained with an anti-vinculin antibody. Scale bar: 50 um; the insets show a higher magnification of the outlined regions. (C) and (D) Zyxin-null fibroblasts were cotransfected with Tes FL-GFP and the constructs DsRed (control), Zyx FL WT-DsRed or Zyx FL MT-DsRed. 24 hours after transfection, cells were fixed and stained for vinculin, and analyzed by immunofluorescence confocal microscopy. Measured intensities at FAs were then averaged for each cell. Bar plots indicate the intercellular means of measured intensities of Tes FL-GFP (C) and the means of the integrated fluorescence intensities of Zyx FL WT-DsRed and Zyx FL MT-DsRed (D) For all quantifications at least 25 cells and >1500 FAs per condition were analyzed. Error bars represent S.E.M. *** P<0.0001, only significant differences are indicated.

We attempted to reproduce the above results (represented in Fig 5B) with full-length Tes, and unexpectedly, we observed that endogenous Tes, as well as Tes FL GFP, could be recruited to FAs in zyxin-null fibroblasts (Fig 6A and 6B) indicating that this recruitment can also occur independently of zyxin. These results together with those obtained for GFP-Tes LIM1 demonstrate that in fibroblasts, full-length Tes can also be recruited to FAs in a zyxin-independent way and that this recruitment does not seem to involve Tes LIM1. Furthermore, we monitored whether variations in zyxin expression levels influence Tes expression and, reciprocally, whether loss or overexpression of Tes affects zyxin expression levels. To this end, MEF cells were transfected with siRNAs directed against Tes or zyxin respectively. Quantitative analysis of Western blot bands did not reveal any significant differences (S2 Fig). Similarly, overexpression of GFP-zyxin or GFP-Tes did not reveal any effect on the expression levels of the endogenous proteins.

Although the zyxin-independent recruitment of Tes complicates analysis, we quantitatively assessed the impact of the zyxin-Tes interaction on the localization of both proteins to FAs by using immunofluorescence microscopy. To this end we transfected zyxin-null fibroblasts with expression constructs for Tes FL-GFP and Zyx FL WT-DsRed, the variant Zyx FL MT-DsRed, or DsRed (control). After fixation, cells were stained with an antibody for the FA marker vinculin. FA characterization was only carried out for cells expressing both GFP and DsRed constructs. Corroborating our results above, Tes quantities in FAs were enhanced (twofold) in the presence of Zyx FL WT compared to control, but not in the presence of Zyx FL MT (Fig 6C). Tes FL-GFP quantities at FAs in case of the presence of Zyx FL MT were comparable to the control zyxin-null cells (Fig 6C) and likely represent the zyxin-independent recruitment to FAs. The results indicate that mutation of VGEI abolishes the interaction between Tes FL and zyxin in FAs. Notably, the levels of Zyx FL WT and Zyx FL MT in FAs were not statistically different (Fig 6D), indicating that zyxin recruits Tes to FAs, but not vice versa. Taken together, these findings demonstrate that the VGEI-mediated zyxin-Tes interaction increases Tes quantities in FAs.

### Interaction between zyxin and Tes modulates kinetics of Tes at FAs

**Fig 7:**
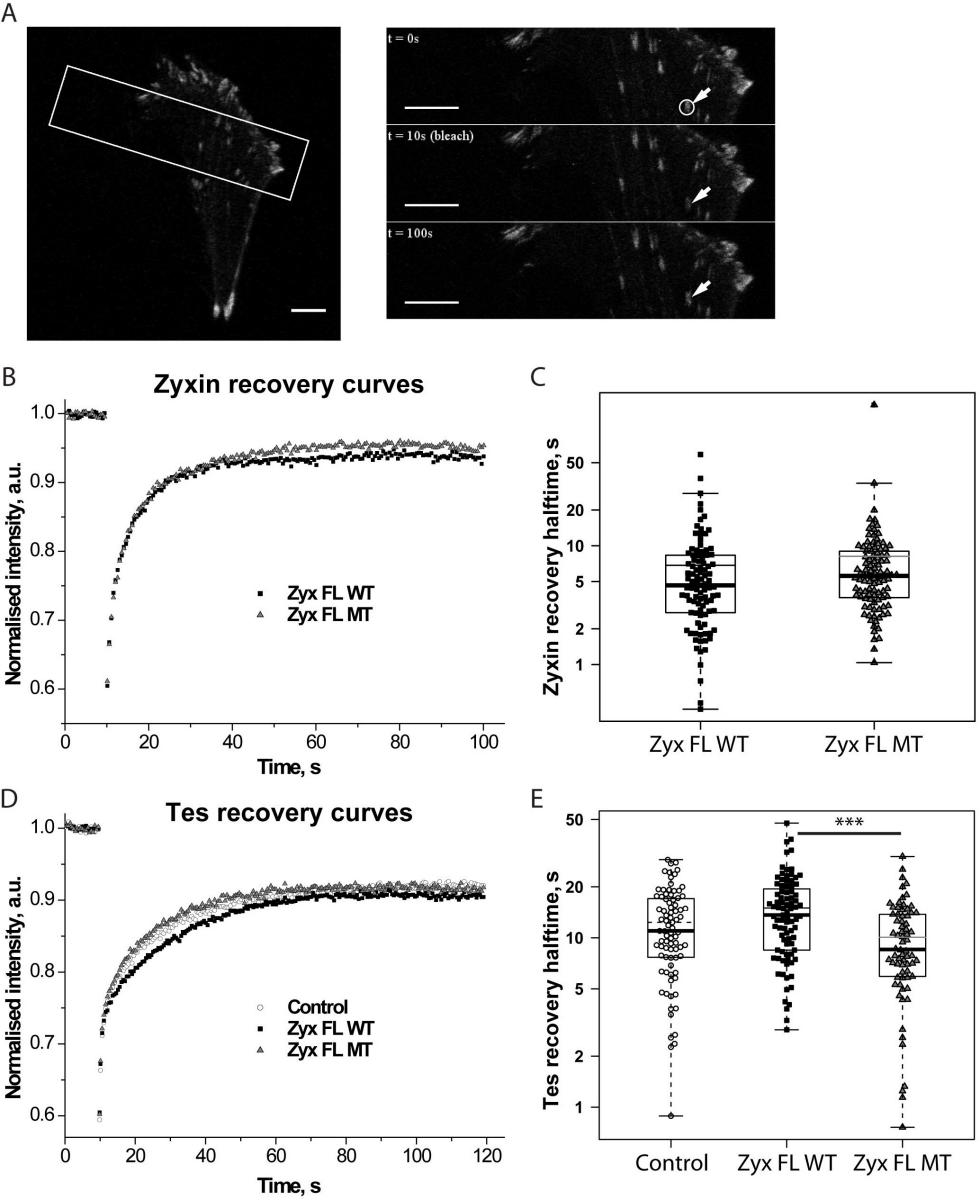
Dynamics of zyxin and Tes at FAs. (A) A typical FRAP experiment with Zyx FL WT-GFP in zyxin-null fibroblasts. Left panel: A cell expressing Zyx FL WT-GFP. The white rectangle indicates the area selected for FRAP acquisition. Right panels represent the time-course of a FRAP experiment: prebleach image (top), first image after bleach (middle), 100 s after start of acquisition (bottom). The white circle outlines the region selected for the bleach. Scale bars: 10 μm. (B) Normalized and averaged Zyx FL WT-GFP (Zyx FL WT) and Zyx FL MT-GFP (Zyx FL MT) recovery curves obtained from four independent experiments (in total about 100 acquisitions per condition). (C) *t*_*½*_ values of recovery halftimes of individual zyxin-GFP FRAP recoveries that were averaged in B are represented as Box-and-Whisker plots overlaid with data points. Thin lines inside boxes represent mean *t*_*½*_ values. (D) Normalized and averaged Tes FL-GFP recovery curves in the presence of DsRed (Control), Zyx FL WT-DsRed (Zyx FL WT) or Zyx FL MT-DsRed (Zyx FL MT) obtained from four independent experiments (in total about 80 acquisitions per condition). (E) *t*_*½*_ values of recovery halftimes of individual Tes FL-GFP FRAP recoveries that were averaged in D are represented as Box-and-Whisker plots overlaid with data points. Thin lines inside boxes represent mean *t*_*½*_ values. *** P<0.0001, only significant differences are indicated.

To determine if a loss of interaction between Tes and zyxin influences the turnover of each of these proteins at FAs, we performed FRAP experiments (Fig 7A). We first investigated the effects of the zyxin-Tes interaction on zyxin turnover in FAs. To this end, we transfected zyxin-null fibroblasts with Zyx FL WT-GFP and Zyx FL MT-GFP and measured turnover times of these proteins at FAs by FRAP (Fig 7B and 7C). The individual fluorescence recoveries of both Zyx FL WT and Zyx FL MT at FAs could be well fitted by a single exponential function. The performed analysis did not reveal any significant increase or decrease of recovery halftimes (*t*_*½*_) for Zyx FL MT in comparison to Zyx FL WT (p-value ≈ 0.1) (Fig 7C), in line with the observation that Tes does not recruit zyxin to FAs. However, the variation of individual halftime values was significantly larger for Zyx FL WT in comparison with Zyx FL MT as shown by the Fligner-Killeen test of homogeneity of variances (p-value < 0.05, see Materials and methods) suggesting that the heterogeneity of zyxin dynamics in the analyzed FA population is higher when the zyxin-Tes interaction is intact.

As Tes can be recruited to FAs in the absence of zyxin, we decided to analyze binding kinetics of Tes in FAs in three different conditions: in the absence of zyxin (DsRed), in the presence of Zyx FL WT-DsRed or in the presence of Zyx FL MT-DsRed (Fig 7D and 7E). In each case FRAP of the Tes FL-GFP was used to determine the kinetics of Tes in FAs. Only curves that fitted with a single exponential function were retained (≈70% of the curves). Recovery of Tes was generally slower than for zyxin (compare panels C and E). The average *t*_*½*_ value for Tes in the presence of wild-type zyxin (13.5 seconds) at FAs was approximately threefold higher than the corresponding average *t*_*½*_ for wild-type zyxin (4.8 seconds), indicating that the exchange of zyxin in FAs is much faster than the exchange of Tes. Furthermore, the statistical analysis revealed that recovery of Tes in the presence of Zyx FL MT was significantly faster than in the presence of Zyx FL WT (Fig 7D and 7E). Surprisingly, in cells that were not transfected with any zyxin construct, the average recovery *t*_*½*_ of Tes FL-GFP at FAswas only slightly lower than in Zyx FL WT expressing cells and slightly higher than in Zyx FL MT expressing cells, resulting in no statistically significant differences of the average recovery halftime of Tes between control cells and Zyx FL WT or Zyx FL MT cells (Fig 7E).

### Zyxin affects FA numbers and lifetime independently of Tes

**Fig 8:**
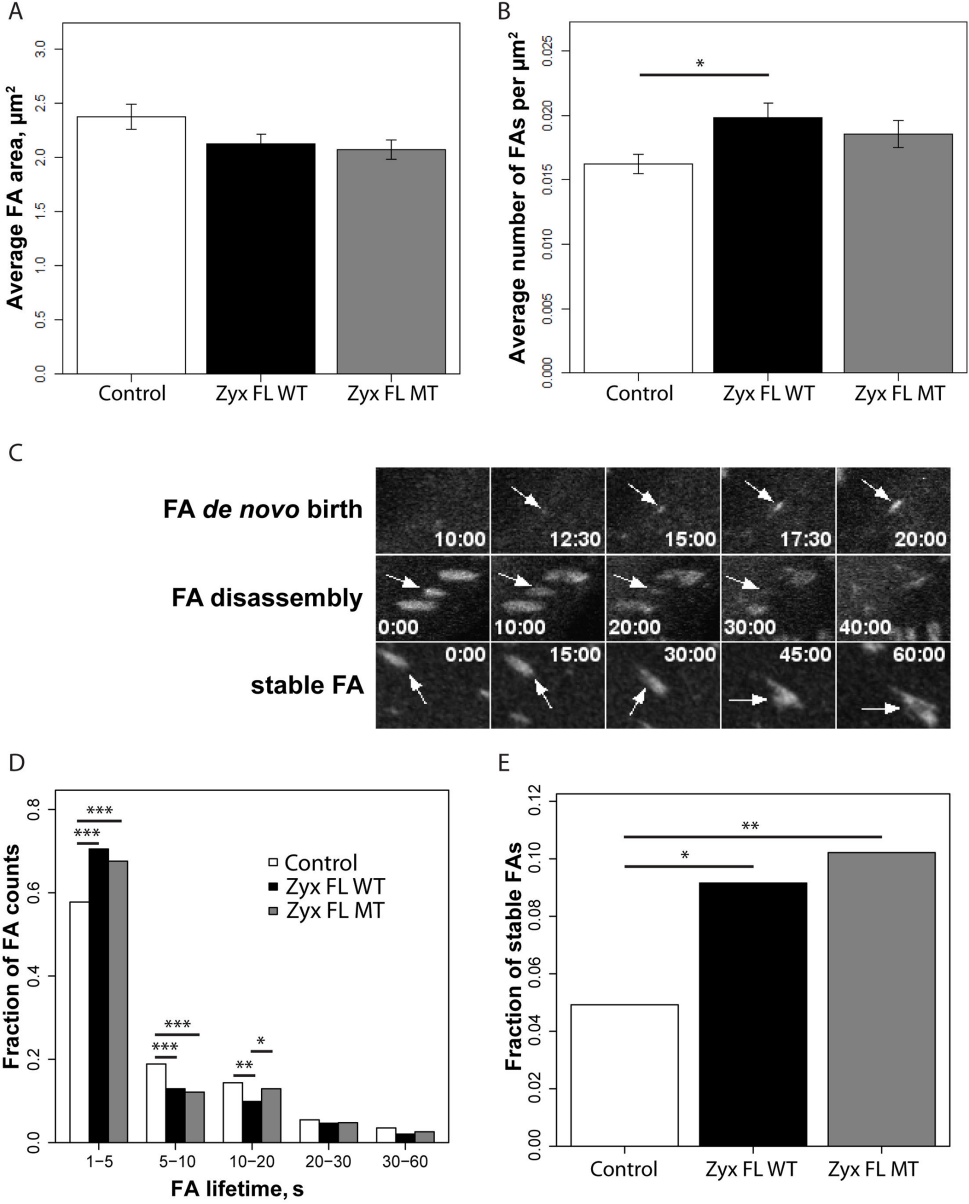
Zyxin affects FA density in cells, FA morphology and FA lifecycle in a Tes-independent manner. (A) Average FA size in the presence of DsRed (Control), Zyx FL WT-DsRed (Zyx FL WT) or Zyx FL MT-DsRed (Zyx FL MT). (B) Average number of FAs per μm^2^ of cell area in the presence of DsRed (Control), Zyx FL WT-DsRed (Zyx FL WT) or Zyx FL MT-DsRed (Zyx FL MT). In (A) and (B) measurements are based on vinculin staining with an anti-vinculin antibody and were first averaged per cell, barplots represent means ± S.E.M. of these values. For quantifications in A and B at least 25 cells and >1500 FAs per condition were analyzed. (C) Time-course panels representing different stages of FA lifecycle followed using mEmerald paxillin: FA de novo birth (upper panels), FA disassembly (middle panels), and stable FA existing throughout the observation time period (lower panels). Despite of having long lifetimes, these FAs can shift position and substantially change their phenotype. (D) Histogram representing the distribution of FA lifetimes in the presence of DsRed (Control), Zyx FL WT-DsRed (Zyx FL WT) or Zyx FL MT-DsRed (Zyx FL MT). 1023, 1618 and 1006 tracks accordingly were considered (see Materials and methods for details). (E) Fraction number of stable FAs (25, 65 and 51 tracks for Control, Zyx FL WT and Zyx FL MT respectively) presented as fractions relative to the average number of FAs per time frame. For quantifications in (D) and (E) at least 10 cells and >1000 FAs per condition were analyzed. *P<0.05, **P<0.01, ***P<0.0001, only significant differences are indicated.

We next tested whether the Tes-zyxin interaction affects FA properties. To this end, zyxin-null fibroblasts were transfected with expression constructs for Zyx FL WT-DsRed or the variant Zyx FL MT-DsRed, using DsRed expressing cells as control, and stained with an antibody directed against the FA marker vinculin. FA characterization was only carried out in DsRed positive cells. FA size was not influenced by the expression of Zyx FL WT or Zyx FL MT (Fig 8A). However, compared to control cells, expression of Zyx FL WT led to a significant increase (≈22%) in FA number per cell area unit (Fig 8B). FA number in cells expressing Zyx FL MT was in between that in control and Zyx FL WT cells but no statistically significant difference could be determined. Additionally, actin staining revealed that the reintroduction of Zyx FL WT and Zyx FL MT enhanced the quantity of actin in FAs compared to control (S3 Fig). Interestingly, overexpression of Tes GFP in zyxin-null cells also increased the number of FAs while a knockdown of Tes led to a decrease in FA numbers (S4 Fig).

The higher amount of Tes in FAs of Zyx FL WT expressing cells (Fig 6C) correlates with the increase in FA number per area unit. This suggests that the zyxin-Tes interaction may take part in the regulation of FA dynamics and their lifetimes. To investigate this, we determined whether the interaction between zyxin and Tes modulates the turnover of FAs. Zyxin-null fibroblasts were co-transfected with mEmerald-paxillin and Zyx FL WT-DsRed, Zyx FL MT-DsRed or DsRed (control). Transfected cells were imaged during 1 hour by time-lapse microscopy. To determine FA lifetimes, their assembly/disassembly events were detected using paxillin as FA marker (Fig 8C, 8D and 8E). The majority of FAs were dynamic (Fig 8D) and only a small population (<10%) was stable during the whole acquisition period (Fig 8E). We considered FAs as being stable, when they were present at the start of the acquisition and existed throughout the observation period. Expression of Zyx FL WT or Zyx FL MT into zyxin-null fibroblasts led to an increase of the number of stable FAs (Fig 8E). The distribution of FA lifetimes in the dynamic FA population shows that Zyx FL WT and Zyx FL MT increased the fraction of dynamic FAs with a short lifetime (1–5 min, representing ~60% of total and ~70% in zyxin-expressing cells) while decreasing the fractions of dynamic FAs with longer lifetimes (Fig 8D). Overall, no statistically significant differences were identified between Zyx FL WT and Zyx FL MT. Thus, the expression of both Zyx FL WT and Zyx FL MT decreased the FA lifetime in comparison to control cells. These data suggest that zyxin promotes turnover of dynamic FAs, but does so independently of Tes.

As both zyxin and Tes have been proposed to regulate the actin cytoskeleton, we chose to investigate the turnover of mCherry-actin in the presence of Zyx FL WT-GFP or Zyx FL MT-GFP. The slow turnover of actin (longer acquisition times) and the highly dynamic nature of the actin cytoskeleton translated into FAs which were significantly moving and/or were undergoing assembly/disassembly processes during the acquisition time, rendered the evaluation difficult. We used the zyxin constructs as markers to track this FA movement. FAs undergoing notable assembly/disassembly processes during the acquisition (~70%) were not selected for evaluation resulting in a low number of usable acquisitions. No statistically significant difference between the turnover rates of mCherry-actin in the presence of Zyx FL WT or Zyx FL MT were determined (S3 Fig).

### The interaction between zyxin and Tes influences cell spreading

**Fig 9:**
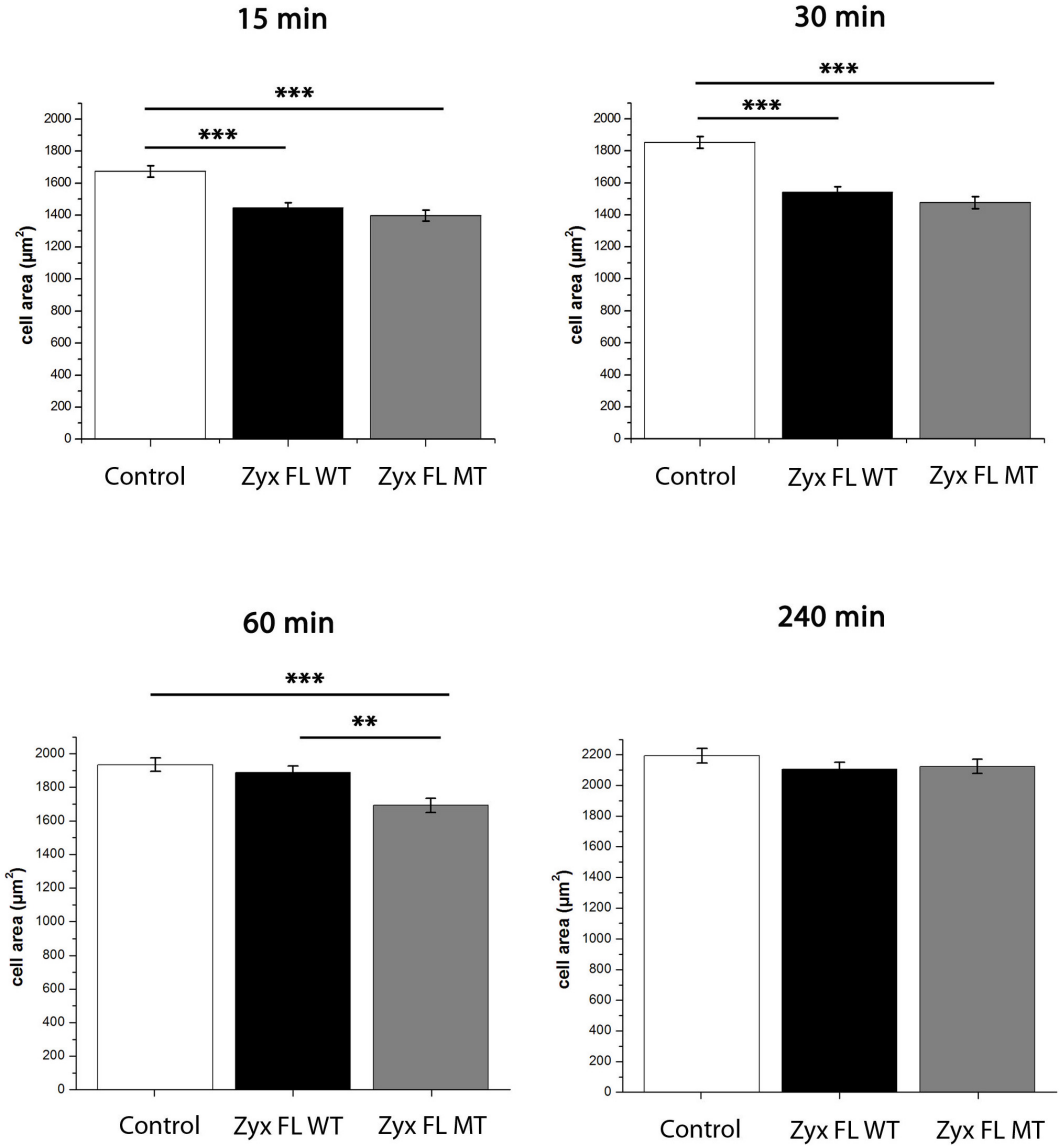
Tes interaction with zyxin affects cell spreading. Zyxin-null fibroblasts were transfected with GFP (Control), Zyx FL WT-GFP or Zyx FL MT-GFP. Spreading assays were performed as described in Materials and methods. Cell surfaces of individual transfected cells were determined 15 min, 30 min, 60 min, and 240 min after plating on fibronectin (20 μg/ml). Bar plots correspond to the means ± S.E.M. of 3 independent experiments. ** P<0.01, *** P<0.0001, only significant differences are indicated.

As Tes and zyxin have both been shown to play a role in cell spreading, we wanted to assess the importance of their interaction in the regulation of this process. We analyzed this by comparing cell areas of zyxin-null fibroblasts transfected with Zyx FL WT-GFP or with Zyx FL MT-GFP. Resuspended cells were plated onto coverslips coated with fibronectin and allowed to adhere either for 15, 30, 60 or 240 minutes. During cell spreading, both zyxin variants localized to FAs and to a lesser extent to stress fibers. Quantitative analysis of the time points 15, 30 and 60 minutes revealed that cells expressing constructs Zyx FL WT or Zyx FL MT, displayed a smaller cell surface compared to control cells. Additionally, the expression of the Zyx FL MT construct in zyxin-null fibroblasts, further altered their spreading on fibronectin at the 60 minutes time point (Fig 9). Indeed, Zyx FL MT cells showed a decrease of the average surface as compared to cells expressing Zyx FL WT, indicating that the cells expressing Zyx FL MT were impaired in their spreading ability to a stronger extent than Zyx FL WT expressing cells. At a later time point (240 minutes) statistically relevant differences between the three conditions were no longer observed (Fig 9B). These results indicate that zyxin affects cell spreading, and that the interaction between Tes and zyxin appears transiently involved in the process of cell spreading.

## Discussion

In this study we have delineated the Tes binding site within zyxin to a sequence comprising zyxin residues 51–63 and we have shown that mutating the amino acids VGEI contained in this region was sufficient to abolish the interaction. Analysis of the variant Zyx FL MT in cells revealed that zyxin influences FA dynamics independently of Tes suggesting that zyxin acts upstream of Tes. Nevertheless, we have shown that the interaction between zyxin and Tes is functional since it enhances recruitment of Tes to FAs and it promotes the kinetics of cell spreading.

### Identification of the Tes binding site in zyxin

The binding site of zyxin within Tes has previously been described [20], and corresponds to the first LIM domain of Tes. To determine the complementary binding site of Tes in zyxin, we combined ectopic recruitment and GST pull-down experiments and we showed that the first LIM domain of Tes is capable of interacting with residues 51–63 of zyxin. Moreover, mutation of the four conserved amino acids VGEI within this region in full-length zyxin abolished Tes binding, indicating that the VGEI sequence is crucial for the interaction. Our results illustrate that the binding site comprised within residues 51–63 is necessary and sufficient to bind Tes. The sequence seems to be unique to zyxin, as sequence alignments did not identify it in other eukaryotic proteins including the closest zyxin homolog, lipoma preferred partner (LPP). The binding site of Tes in zyxin is located between the binding sites of α-actinin and Ena/VASP proteins [16]. Indeed, the binding sites of Tes and Ena/VASP proteins are adjacent, as the VGEI motif and the first FPPPP motif are only separated by 7 amino acids, while the distance between the binding sites for α-actinin (first 50 residues) and for Tes is similarly small. Furthermore, Tes has been described to interact with VASP and Mena through its LIM domains and with α-actinin through its N-terminal region (residues 1–234) [20, 24]. Given that the three binding sites in zyxin are in such close proximity, it raises the interesting possibility that these proteins could interact with each other and perhaps form various supramolecular complexes active in FAs. However this question remains to be elucidated.

### Zyxin and Tes at focal adhesions, who is influencing who?

Our new observation of zyxin-independent recruitment of Tes to FAs and the potential existence of different ternary or quaternary complexes containing zyxin and Tes obviously complicates the interpretation on the measured dynamic properties of zyxin and Tes and their interplay at FAs.

Whereas zyxin does not influences Tes expression levels (and vice-versa), zyxin positively affects Tes quantities in FAs as disruption of their interaction reduces the amount of Tes by twofold. The recovery halftime of Tes, measured by FRAP, was significantly higher in the presence of Zyx FL WT as compared to Zyx FL MT (Fig 7D and 7E). Combined these findings indicate that zyxin can stabilize Tes in FAs. In the presence of Zyx FL MT, the enhanced Tes turnover rates could result solely from the zyxin-independent recruitment whereas the recovery curves in the presence of Zyx FL WT could represent the turnovers of two Tes populations. Thus, it may well be that the bleached Tes molecules exchange more slowly in a scaffolding interaction with zyxin, whereas in the case of zyxin-independent recruitment the kinetics of Tes appear to be faster. Obviously, the differences in *t*_*½*_-values of Tes reflect differences in the molecular context of the scaffolds recruiting Tes. It is known that FAs correspond to complex protein networks with a large number of possible interactions [30] that may change composition upon maturation [31].

The FRAP recovery halftimes of Tes (average 13.5 seconds) and zyxin (average 4.8 seconds) seen in time-lapse imaging are typical for FA proteins [32, 33]. The different values make a scenario where zyxin-Tes complexes dissociate from or assemble to a FA as a single complex unlikely. In FRAP experiments a slower recovery usually implicates a lower k_off_. Because zyxin recruits Tes, it is therefore somewhat surprising that the exchange of zyxin at FAs is faster than that of Tes suggesting that they have different dissociation rates and that upon zyxin dissociation from the FA it leaves Tes behind. Tes has then to be stabilized by other interactions at FAs and this is consistent with our proposal that (different) zyxin-Tes containing higher order structures in FAs might exist as mentioned above. This does not exclude a scenario in which the zyxin-associated population of Tes (in part) associates with the protein that recruits Tes independently from zyxin, prior to Tes dissociation from FAs.

Tes also appears to contribute to zyxin turnover. Determination of zyxin turnover rates by FRAP revealed that a lack of interaction with Tes resulted in a more narrow distribution of *t*_*½*_ values. It appears that lack of interaction with Tes makes the zyxin behavior with respect to turnover more uniform suggesting more heterogeneity in behavior when Tes is bound to zyxin. This could again involve the formation of different supramolecular complexes on zyxin that directly affect in a positive or negative manner the turnover rates of zyxin.

### Zyxin influences FA properties independently of Tes

Roles for Tes and zyxin in the regulation of the actin cytoskeleton have been previously documented [16–18, 22]. To determine if the interaction between both proteins influences FAs, we probed the effect of a loss of their interaction on the properties of FAs. In zyxin-null cells expression of Zyx FL WT did not result in a difference in the average FA size, but the number of FAs was enhanced. Furthermore, actin quantities in FAs were enhanced in the presence of zyxin. Results obtained with wild-type and mutant zyxin, however, were not statistically different suggesting that this effect is independent of Tes. Similarly, overexpression and knockdown experiments in zyxin-null fibroblasts showed that Tes is capable of regulating FA numbers independently of zyxin. Therefore it seems that Tes and zyxin act, at least partially, independently to influence FA numbers in zyxin-null fibroblasts. Additional analysis of FA lifetimes revealed that wild-type and mutant zyxin both decreased average FA lifetimes by enhancing the number of FAs with a short lifetime. In parallel, wild-type and mutant zyxin enhanced the number of FAs that were stable throughout the 60 minutes observation time. Although this may at first appear contradictory, it may reflect a different effect of zyxin during nascent and mature adhesion formation. Indeed these separate effects of zyxin can reflect the high heterogeneity of FAs. The molecular constitution and morphology of FAs will vary over time as they undergo assembly, disassembly and maturation processes [1, 34, 35]. From our analysis, it appears however that the zyxin-Tes interaction is not involved in this and/or that the effects of disrupting the zyxin-Tes interaction are too subtle to be measured with our approach.

### Zyxin-Tes interaction influences cell spreading

Zyxin and Tes have each been described to play a role in cell spreading. Overexpression of Tes has been reported to lead to an increase of the initial cell spreading rate in rat fibroblasts and chicken embryonic fibroblasts, while not affecting the cell surface after spreading. Melanoma cells in which zyxin is upregulated were more spread [36] while the spreading rate on fibronectin was reduced in epithelial cells where zyxin mislocalization was induced by a peptide inhibitor [12]. Furthermore, a knockdown of zyxin has been reported to slow down the spreading rate of MDCK cells on E-cadherin [37]. To investigate if zyxin and Tes cooperate during cell spreading, we performed spreading assays involving Zyx FL WT and Zyx FL MT. To our surprise cells expressing Zyx FL WT presented smaller surfaces at the time points 15 min and 30 min than control cells while after 60 min reintroduction of Zyx FL WT did not affect the spreading rate of zyxin-null fibroblasts on fibronectin. These results contrast with previous findings obtained with PtK2 epithelial cells and microvascular smooth muscle cells [8, 12] showing that zyxin promotes cell spreading. One possible explanation is that the effect is cell type dependent. This is not without precedence as different cell lines have been shown to spread differently on the same surface [38]. Furthermore, whereas zyxin has been shown to promote cell spreading, it has also been shown to inhibit cell adhesion [8]. As the processes of cell adhesion and cell spreading are closely linked and cells transition from the process of cell adhesion to the processes of cell spreading, the reduced cell surface at the time points 15 and 30 min could be the result of a delayed start of the spreading mechanism due to an inhibition of the cell adhesion process by the reintroduction of zyxin. Another likely possibility is that zyxin is not the sole determinant of the spreading rate. Indeed our results with Zyx FL MT show that a lack of interaction between zyxin and Tes reduces the spreading rate of zyxin-null fibroblasts after 60 minutes, while after 15 and 30 min and 4 hours no difference could be determined, indicating that Tes and zyxin interact to regulate an intermediate phase of cell spreading. The mechanism by which Zyx FL MT decreases the cell spreading rate remains to be clarified. A possibility is that a lack of interaction between Tes and zyxin leads to the recruitment of another partner which negatively impacts cell spreading. Indeed different pathways and proteins which inhibit cell spreading have already been described [39–42]. Of particular interest, Nishiya and colleagues described that Hic-5 inhibits integrin-mediated cell spreading of NIH 3T3 cells on fibronectin by competing with paxillin for FAK. A similar mechanism could account for the observation regarding our effects of zyxin and Tes on spreading. Drees *et al*. demonstrated that a perturbation of the interaction between zyxin and members of the Ena/VASP family similarly reduces cell spreading on fibronectin [16]. Mena and VASP have also been shown to interact with Tes and, given that the Tes binding site is adjacent to FPPPP-motifs in zyxin, these proteins may exist in one complex in FAs as has been proposed before [20, 21]. Taken together, this protein complex may participate in the regulation of cell spreading and the elimination of one partner from the complex may reduce the cell spreading rate.

In summary, our findings identify the interaction site of Tes in zyxin which is located between the α-actinin and the VASP binding sites. We show that zyxin recruits Tes to FAs although in fibroblasts there appears to be a portion of Tes molecules that localize to FAs independently of zyxin. Furthermore, our data also imply that through their interaction at FAs, zyxin and Tes act together to regulate cell spreading and that zyxin influences FA dynamics.

## Supporting Information

S1 Fig VASP and α-actinin are still recruited by zyxin variants lacking the VGEI motif. A) Schematic representation of the first 140 amino acids of Zyxin containing the α-actinin (AcBS, green) and VASP (FPPPP, red) binding sites as well as the VGEI motif (VGEI, yellow). B) Zyxin-null fibroblasts transfected with Zyx FL WT -mito and Zyx FL MT- mito. Zyxin-mito constructs were labelled with a rabbit anti-zyxin mAb, VASP with a mouse anti-VASP mAb and α-actinin with a mouse anti-α-actinin mAb. Zyx FL WT -mito as well as Zyx FL MT -mito recruited VASP and α-actinin to the surface of mitochondria. Scale bar: 50 μm.
(TIF)

S2 Fig Variation of Tes levels do not affect zyxin levels and vice versa. (A) Representative Western blot analysis and quantification of the expression of endogenous zyxin or endogenous Tes in MEF cells transfected with control siRNA (control), siRNA against Tes (siRNA Tes), or siRNA against zyxin (siRNA Zyx). GADPDH was used as a loading control. The graphs on the side of each blot show the respective quantification of proteins levels after normalization to GAPDH signal (n = 3) (B) Representative Western blot analysis and quantification of the expression of endogenous zyxin or endogenous Tes in MEF cells transfected with GFP, Tes FL-GFP, or Zyx FL WT-GFP. GAPDH was used as a loading control. The graphs on the side of each blot show the respective quantification of proteins levels after normalization to GAPDH signal (n = 3). For (A) and (B), data represent the mean of three independent experiments; error bars indicate S.E.M.
(TIF)

S3 Fig Loss of interaction between zyxin and Tes does not have a significant effect on the measured actin kinetics at FAs. (A) Normalized and averaged mCherry-actin recovery curves in presence of Zyx FL WT-GFP (Zyx FL WT) or Zyx FL MT-GFP (Zyx FL MT) obtained from three independent experiments (in total 17 acquisitions in case of Zyx FL WT and 15 acquisitions in case of Zyx FL MT). Only two conditions were compared in this experiment, because zyxin signal was used to segment the bleached FAs and track their positions during the recovery time-course (see Materials and Methods section of the manuscript for the details). (B) Halftimes of individual mCherry-actin FRAP recoveries are represented as Box-and-Whisker plots overlaid with data points. Thin lines inside boxes represent mean halftime values. Although we did not identify statistically significant differences for the recovery halftimes (the Mann-Whitney U test was used), it does not mean that the studied interaction has no effect on actin kinetics. Importantly, we were able to acquire representative recoveries only for relatively long-living FAs, which do not represent the majority of FA population (see Fig 8D in the main text). FAs which underwent noticable assembly or disassembly processes during the recovery acquisition were discarded from the analysis. (C) Histograms indicate the fluorescence intensities of actin. For all quantifications at least 25 cells and >1500 FAs per condition were analyzed. Error bars indicate S.E.M. *P<0.05,***P<0.0001.
(TIF)

S4 Fig Variation of Tes levels influence the number of FAs independently of zyxin. (A) Average number of FAs per μm2 of cell area in the presence of GFP (Control), or Tes FL-GFP (Tes FL). (B) Average number of FA per μm2 of cell area in the presence of control siRNA (Control) and siRNA directed against Tes (siRNA Tes). In (A) and (B) measurements are based on vinculin staining with an anti-vinculin antibody and were first averaged per cell. For quantifications in A and B at least 25 cells and >1500 FAs per condition were analyzed. Barplots represent means ± S.E.M. of these values. *P<0.05,**P<0.005.
(TIF)
